# Effects of Grinding Methods and Water-to-Binder Ratio on the Properties of Cement Mortars Blended with Biomass Ash and Ceramic Powder

**DOI:** 10.3390/ma16062443

**Published:** 2023-03-18

**Authors:** Vladan Pantić, Slobodan Šupić, Milica Vučinić-Vasić, Tomas Nemeš, Mirjana Malešev, Ivan Lukić, Vlastimir Radonjanin

**Affiliations:** Department of Civil Engineering and Geodesy, Faculty of Technical Sciences, University of Novi Sad, 21000 Novi Sad, Serbia

**Keywords:** corn cob ash, ceramic powder, mortar, SCM, dilution effect, water-to-binder ratio

## Abstract

To combat environmental challenges—such as the depletion of natural resources and a high carbon footprint—and contribute to the effort of achieving zero-waste technology and sustainable development, the use of agricultural and industrial wastes in the cement industry has created a research interest. This study explores the potential of two types of harvest residue ash (HRA) and three types of ceramic waste (CP) as supplementary cementitious materials (SCMs) through: (1) the characterization of raw materials and (2) examining the physical properties and mechanical performance of cement-based mortar samples prepared with 10%, 30% and 50%wt of the selected SCMs ground into powder form as cement replacement. Two main variables were the water-to-binder ratio (w/b) and the effect of different grinding procedures. Experimental results demonstrated that flexural and compressive strengths were not significantly impaired by SCM additions of up to 50%, but higher replacement levels led to an increased permeability and higher capillary water absorption due to the dilution effect. Also, a lower w/b was shown to effectively reduce the porosity of mortar and increase its mechanical properties, allowing for higher shares of SCMs to be utilized. This study verifies the technical feasibility of cob corn ash and ceramic powder application as SCMs in mortar formulations, further promoting the practice of incorporating industrial and agricultural by-products in greener cementitious composites.

## 1. Introduction

The cement industry is one of the leading contributors to the carbon footprint, and the production of Portland cement (PC) accounts for 10–23% of total anthropogenic CO_2_ emissions [1,2], resulting from (1) carbonate decomposition and (2) the oxidation of fossil fuels. The minimum theoretical energy required for calcination is estimated to be about 1760 MJ/t clinker, while some modern plants consume around 2800 MJ/t clinker [3]. Considering the everlasting global energy crisis, the use of alternative fuels is now an imperative in many countries.

Substituting clinker with pozzolanic (e.g., silica fume) or latent hydraulic (e.g., ground granulated blast-furnace slag (GBFS)) materials allows for reducing the proportion of clinker in cement. However, some of the most used supplementary cementitious materials (SCMs) are dwindling: fly ash in the wake of coal replacement with renewables and GBFS by virtue of the recycling process use in steel production rather than iron ore. In a bid to adhere to the principles of sustainable development and reduce carbon emissions, countries all over the world have been shutting down coal thermal power plants and making a shift towards renewable energy sources, such as biomass. As a result of the rapid establishment of biomass-based power plants, the disposal of agro-waste ashes has increased manifold and poses a severe challenge to the environment. The substitution of PC with an alternative binding material, such as biomass ash, will reduce the environmental effects in terms of the amount of energy used, pollution and waste management, simultaneously addressing the problem of climate change.

Growing quantities of harvest residue ash (HRA) are created as waste products during the combustion, pyrolysis and gasification of agricultural biomass in Vojvodina— the northern province of Serbia—currently estimated at 5000 tons per year [4]. These ashes are most commonly disposed of in open landfills or recycled on agricultural farms. Several investigations have been attempted by a number of researchers to develop alternative cementitious materials using HRA. Rice husk ash (RHA) [5], palm oil fuel ash (POFA) [6], sugar cane bagasse ash (SCBA) [7], wheat straw ash (WSA) [8] and corn cob ash (CCA) [9] are some of the HRA that have evidently been reported for their suitability as pozzolans. The majority of the agricultural biomass resources in Serbia lie in corn biomass, more than 25% in the straw of cereals, and the rest in harvest residues of sunflower, soybean and oilseed rape. The cataloging of the available harvest residue ashes in Serbia has been conducted, and the results of testing their basic chemical and physical properties have been reported in [10]. Exploring the possibility of the application of HRA as SCMs in mortar production in Serbia has been recently initiated. Few earlier studies are available on the effect of WSA incorporation on the flexural and compressive strength of blended cement mortar [11] and masonry mortar [8]. No studies referring to CCA application in cement-based composites in Serbia can be found, while there have been studies giving an account of this by-product utilization as a SCM in other countries [9].

The other type of refuse that is reviewed in this study is ceramic waste. A large quantity of ceramic industry by-products is produced during the manufacture of ceramic products and the demolition of buildings. The total amount of ceramic waste generated from the production steps of ceramic factories is about 3–7% of their final production [12], which indicates substantial portions of tiles and ceramic waste annually. Three major ceramic industry companies from Vojvodina generate over 6000 tons of ceramic waste per year. A literature review has confirmed that ceramic waste can replace fine natural aggregates in concrete and mortar [13,14]. Finely ground ceramic powder (CP) may be a promising material for replacing cement, which could gain environmental benefits by increasing construction waste consumption and reducing CO_2_ emissions. The application of CP in cement-based composites has been investigated to a limited extent. Lasseugette et al. [15] reported that there is no detrimental effect on the mechanical properties of mortar with no more than 15% CP as a cement substitute. Considering its application in concrete, several authors [16,17] have suggested that high-performance concrete can be produced with the replacement of up to 40% of PC with CP without compromising its mechanical and durability properties. No recommendations of a higher portion of PC with CP in cementitious systems have been recorded.

From the earlier research studies [18,19], it was observed that different processing methods have a remarkable influence on the pozzolanic performance of industrial and agricultural by-products. Processing methods—such as sieving or grinding in a mill—help turn them into highly reactive pozzolans, owing to the availability of a greater number of nucleation sites for the cement hydration to take place. Rukzon et al. [20] stated that an increased level of fineness leads to a progressive increase in the packing density—the filling of interstices between coarse particles—which is an additional reason for the strength enhancement. Most researchers [21,22,23,24] reported using a ball mill for the grinding procedure and suggested an optimum duration (varying from 30 min to 6 h) for various by-products. Based on the literature review, it can be inferred that the highest pozzolanic reactivity can be achieved after grinding a specific SCM for the corresponding optimum duration.

At cement replacement levels higher than 40%, a number of authors described a steady drop in the compressive strength of mortar and concrete that can be attributed to the dilution effect entailing a lower cement quantity available for hydration, a decreased number of hydration products and, consequently, a more permeable matrix that is accountable for reduced strength and higher water absorption. Hence, some authors combined different grinding procedures to analyze the influence of various pre-treatments on the pozzolanicity of ground SCMs and their application as cement substitutes at higher replacement levels. Qudoos et al. [25] coupled a ball mill and a disintegrator mill as milling techniques, while Cordeiro et al. [26] used a combination of vibratory and tumbling mills for various durations to study the pozzolanic efficiency of ground materials.

Instead of combining energy-intensive processing methods, the authors of this paper suggest the approach in which the strength and durability of cementitious composites may be improved by a proper mix design in conjunction with a single grinding procedure. Lowering the water-to-binder ratio (w/b) can be an effective method to mitigate the dilution effect, i.e., to reduce the capillary porosity of the mortar blended with higher shares of SCMs and enhance its durability performance. A lower w/b results in the transformation of larger, connected, capillary pores into smaller ones, further signifying that the transport of water and aggressive chemicals through the newly developed less permeable cement matrix is limited. Hence, it could conceivably be hypothesized that the appropriate grinding procedure and an optimized mix design will make it possible to obtain mortar, blended with ceramic powder and biomass ash, with physical and mechanical characteristics comparable to the reference cement sample. With the aforesaid objections in mind, the use of finely ground HRA and CP in combination with a proper mix design in mortar has been proposed in this research as a good solution for reusing industrial and agricultural waste effectively and producing green cementitious materials. Waste ceramics from three local factories and HRA from two major biomass ash producers in Vojvodina were supplied and ground into powder form using two different grinding procedures. The performances of all the collected and prepared materials were assessed through detailed characterization, which included testing all relevant chemical, mineralogical, physical, mechanical and pozzolanic properties and comparing them with the parameters of the reference PC. The feasibility of the selected SCMs employed as partial substitutes of cement up to 50% to prepare blended cement mortars was evaluated on the basis of their mechanical properties—flexural and compressive strength—and physical properties—capillary water absorption. In addition, lowering w/b proved to be an efficient way of mitigating the negative influence of the dilution effect, further entailing the prospect of utilizing CP and CCA as promising SCMs, up to 50%, to develop sustainable and durable cement-based materials for future engineering applications.

## 2. Materials and Methods

### 2.1. Materials

#### 2.1.1. Cement

Portland cement (PC), originating from Lafarge cement factory in Beočin, Serbia, was used. The cement has a specific gravity of 3.1 g/cm^3^ and Blaine fineness of 4000 cm^2^/g.

#### 2.1.2. Ceramic Waste Powders

Ceramic waste originates from three major ceramic industry companies in Vojvodina: (1) “NEXE-Stražilovo” Petrovaradin, producers of masonry blocks (CP1), (2) “NEXE-Polet” Novi Bečej, roofing tiles manufacturing conglomerate (CP2), (3) “Wienerberger doo” Kanjiža, manufacturers of roofing slipware tiles (CP3).

Two grinding procedures were used: laboratory ball milling technique consisting of a single, uninterrupted grinding process and two-stage milling method involving the use of both laboratory mill and planetary mill with two milling intervals in the latter one.

The samples of ceramic waste (5000 g) were collected and ground in a laboratory ball mill for 6 h, using stainless steel balls, until obtaining a satisfactory level of fineness (the first stage of processing).

In order to assess the possibility of additional mechanical activation and the influence on the reactivity of the tested material, second–stage processing was carried out in a vario-planetary mill (Fritch Pulverisette 4) using stainless steel balls and bowls of 500 mL. The rotational speed of the main disc was 350 rpm, and the ratio between the rotational speed of the main disc and vials was −3.43. The samples were milled in two 20 min intervals with a 10 min break. The total milling time was 40 min for all the samples.

#### 2.1.3. Biomass Ashes

The samples of biomass ashes, a mixture of wheat straw, sunflower husk and silo waste ash (B1) and a mixture of cob corn and soya straw ash (B2), were collected from two producers in Vojvodina, Agricultural enterprise “Almex-Ipok” in Zrenjanin, and the soybean processing factory “Soya-protein” in Novi Bečej, respectively.

The ashes were roughly sieved through a 4 mm sieve in order to separate the un-burnt straw and other large impurities. Mechanical processing conducted for biomass ashes included both above-mentioned grinding procedures.

#### 2.1.4. Fine Aggregate

Standard CEN sand was used as fine aggregate for the characterization of the selected waste materials and the preparation of blended cement-based mortars.

#### 2.1.5. Superplasticizer

Superplasticizer was used to maintain the required consistency while lowering the effective w/b.

### 2.2. Methods

The characterization of biomass ashes and ceramic powders included several physical and chemical properties tests according to the relevant standards as well as the evaluation of the criteria conformity. The chemical composition was tested using an energy–dispersive X-ray fluorescence spectrometer (EDXRF 2000 Oxford instruments, Belgrade, Serbia) according to EN 196-2 and ISO 29581-2. The representative samples (100 g) were pulverized in a laboratory vibratory mill prior to testing. The loss on ignition (LOI) was determined as a weight difference between 20 °C and 950 °C.

The specific surface area of biomass ashes was determined according to Blaine air permeability method given in EN 196-6, which is widely used for characterizing Portland cement.

Initial and final setting time, fineness and soundness of biomass ashes were defined in accordance with EN 196-3.

The pozzolanic activity was studied in the samples prepared according to the procedure given in SRPS B.C1.018. Mortars were prepared with biomass ash, slaked lime and standard sand, with the following mass proportions: m_sl_:m_bash_:m_qs_ = 1:2:9 and water–binder ratio 0.6 (where: m_sl_—mass of slaked lime; m_bash_—mass of biomass ash; m_qs_—mass of CEN standard sand). After compacting, the samples were hermetically sealed and cured for 24 h at 20 °C, then for 5 days at 55 °C. Subsequently, 24 h period was allowed for the samples’ cooling process to reach 20 °C, followed by compressive and flexural strength tests (Controls, Cernusco, Italy).

The activity index of biomass ashes was examined according to EN 450-1 (Controls, Cernusco, Italy). Activity index is defined as a ratio (in percent) of the compressive strength of standard mortar bars prepared with 75% test cement plus 25% ash by mass, and the compressive strength of standard mortar bars prepared with 100% cement, when tested at the same age. The preparation of standard mortar bars and determination of the compressive strength were carried out in accordance with EN 196-1.

The mineralogical composition of ceramic waste powders and biomass ashes was specified using the X-ray powder diffraction technique (XRD, Rigaku, Tokyo, Japan). The XRD data were collected from the Rigaku SmartLab 3 kW X-ray powder diffractometer with Cu monochromatic radiation (1.5405 Å). The scanning range was 10–45° in 2θ with a step of 0.05° and a scanning speed of 1°/min.

Particle size distributions of SCMs were determined by the laser diffraction method using a Malvern Mastersizer 2000 particle size analyzer (Malvern, Malvern, UK). Mie light scattering theory, incorporated into the analytical procedure of the Mastersizer 2000 instrument, assumes that particles are spheres, and thus, the results obtained for particle size correspond to equivalent sphere diameters. The measurements were performed with an automated dry dispersion unit Scirocco 2000. The selected nozzle pressure of 2 bar resulted in good reproducibility of the results for all the samples.

Sampling, preparation, storage, curing conditions and mechanical properties tests (compressive and flexural strength) of standard prism samples were carried out in accordance with EN 196-1 (Controls, Cernusco, Italy).

The capillary water absorption of the investigated mortars was measured according to ASTM C1585. The method is used to determine the rate of water absorption by measuring the increase in the mass of a sample caused by the absorption as a function of time when only one surface of the sample is exposed to water.

### 2.3. Mixing and Proportioning of Mortars

The experimental study was performed on seven different mortar mixtures. The composition of mixtures was defined in accordance with EN 196-1: ratio (by mass) of cement and aggregate was 1:3, while w/b was 0.5. The reference mortar (C) was prepared with PC as a binder. In the six remaining mortar mixtures, the part of cement was replaced with 10%, 30% and 50% of optimal types of biomass ash and ceramic powder, selected in the characterization phase (B10, B30, B50 and CP10, CP30, CP50), respectively. The labels and quantities of component materials for each mortar mixture along with the values of effective w/b are listed in Table 1.

The effective w/b was calculated according to the k-value concept. The k-value concept, conforming to EN 206-1, permits a SCM to be taken into account by replacing the term “water/cement ratio” with “water/(cement + k × SCM) ratio”. The amount of the SCM that may be taken into account must meet the following requirement: SCM/cement ≤ 0.33 by mass. For the concrete containing cement type CEM I, the permitted k-value is 0.4.

### 2.4. Mixing and Proportioning of Mortars with Decreased w/b Ratio

The second phase of the experimental study included the preparation of six additional blended mortar mixtures with a decreased effective w/b (fixed value of 0.5), and the quantities of cement, SCM and sand kept constant; the water was reduced, while the super-plasticizer was used to achieve the same consistency as the reference cement mortar. The labels and quantities of component materials for this group of mortars are listed in Table 2.

Standard mortar prisms 40 × 40 × 160 mm were cast for the determination of flexural strength, compressive strength and water absorption due to the capillary action of hardened mortar. The storage and curing conditions were adjusted in consonance with relevant standards and the samples were tested at the appropriate age.

## 3. Test Results and Discussion

### 3.1. Characterization of Materials

#### 3.1.1. Chemical Composition

The results of testing the chemical composition of SCMs, after the grinding procedure in a laboratory ball mill, are summarized in Table 3.

The summarized results of chemical properties, requirements in relevant standards, as well as criteria fulfillment are given in Table 4. Criteria evaluation was conducted in accordance with the relevant standards. As the European standard for biomass ashes and ceramic powders has not been established yet, the assessment of properties was executed as stated in EN450-1.

For all ceramic powders, the sum of the major components (SiO_2_, Al_2_O_3_ and Fe_2_O_3_) is more than 80%, which is above the minimum amount required by EN 450-1 (>70%) for the chemical composition of pozzolans. CP1 is characterized by a higher reactive silica content, which suggests a higher potential reactivity. Ceramic powders satisfy all the chemical criteria regarding their potential application in cement-based composites, apart from free lime content. Free lime may trigger undesired expansion and volume instability when used in concrete as a cement replacement. A greater share of free CaO is expected to be spent within hydration, and, in addition, a re-carbonation reaction can take place due to the reaction of CaO and CO_2_ forming CaCO_3_. Notwithstanding this expectation, the durability issues and time-dependent properties of concrete containing SCMs with elevated percentages of free lime should be taken into account.

The results demonstrate that the chemical composition of different HRA has large variations and this is due to the highly variable contents of bulk inorganic matter and different genetic classes of inorganic matter in biomass varieties. B2 contains significant levels of important oxides and satisfies the criterion for amorphous silica content (>25%), while B1 lacks reactive silica, which is expected to influence its pozzolanic activity.

Both types of biomass ash exceed the limit value for the total amount of alkalis, which could trigger an alkali–aggregate reaction (ASR). Alkali–aggregate reaction refers to a deleterious reaction that occurs over time in cement-based composites between the highly alkaline cement paste and the reactive silica in aggregates. According to [27], ASR can be mitigated if any of the four key factors is eliminated: reactive silica, alkali metals, moisture and sufficient pH. Hence, this mechanism should be experimentally verified in cementitious systems incorporating biomass ashes for each type of aggregate.

Chloride ions are known to impair the service life of reinforced concrete (RC) structures, as they can cause chloride-induced reinforcement corrosion. Both types of biomass ash contain higher concentrations of chloride ions, more than the prescribed value given in EN 450-1. Zhao et al. [28] reported that the chloride binding ratio gradually increases with decreasing w/b and blending with SCMs. 

#### 3.1.2. Physical and Pozzolanic Properties of Raw Materials

The physical and pozzolanic properties of raw materials are listed in Table 5.

All tested materials are characterized by a greater level of fineness in relation to cement. It can be noted that, amongst ceramic powders, CP3 has a noticeably lower specific surface area, which could be attributed to: (1) the higher temperature this type of ceramic is produced at and, consequently, denser structure, or (2) the addition of slipware the ceramic is covered with. The higher level of fineness is expected to induce a higher pozzolanic activity.

All ceramic powders and B2 slightly retarded the setting time of cement paste, as expected when it comes to SCMs. Contrastingly, B1 displayed the shortening of the initial setting time below the required minimum of 60 min. This occurrence may be ascribed to a high alkali content in this type of biomass ash. It is likely that the accelerated setting is a false setting, as alkalis could react with CO_2_ (in the atmosphere) to form alkali carbonates (K_2_O_3_). The produced carbonates will further react with Ca(OH)_2_ liberated by the hydrolysis of C_3_S and form CaCO_3_. This product is known to precipitate and induce the rigidity of the paste. Thus, this type of biomass ash should not be used as a replacement for cement or as a concrete additive.

As previously mentioned, the pozzolanic activity depends not only on the amorphous silica content but also on the grain size. The decrease in particle size improves the pozzolanic activity [29]. Therefore, although the reactive silica content in CP1 and CP3 is almost equal, CP1 has a higher pozzolanic class as a result of a considerably higher level of fineness. 

The activity index for all ceramic powders and B2 at the age of 28 days exceeds 75%, which classifies these materials as pozzolans. As expected, all SCMs showed a consistent increase in strength as the curing period elapsed, which is a direct consequence of a delayed pozzolanic reaction between portlandite from the hydration of Portland cement with silica from the SCM and the subsequent C-S-H gel formation. At the age of 90 days, CP1, CP2 and B2 surpassed the strength of the reference cement. Due to the limited amount of available reactive silica in B1, the pozzolanic reaction of this material was less pronounced and the activity index fell short of the required values at both testing ages. 

#### 3.1.3. Influence of Additional Grinding Time on Relevant Properties of Selected SCMs

Different mechanical processing methods, such as grinding, could have a considerable impact on the particle size and reactivity of these materials. The main advantage of additional grinding lies in the possibility of obtaining finer particles that will fill in the voids of the paste and enhance the pozzolanic reactivity of the material. Two milling methods were used in the grinding process: a laboratory ball milling technique consisting of a single, uninterrupted grinding process and a two-stage milling method involving the use of both a laboratory mill and planetary mill with two milling intervals in the latter one. After grinding in a laboratory ball mill, the other milling technique was employed to study the pozzolanic efficiency of the additionally ground SCMs. The chemical, physical and pozzolanic properties of the selected ceramic powder (CP1) and biomass ash (B2) after each milling method used are listed in Table 6.

The results indicate that significantly finer particles were formed in the planetary mill after the grinding of B2, which suggests that an additional breakdown of particles was attained in the latter grinding method. The reduction in the particle size of ceramic powder was less noticeable, as the specific surface area increased by cca 8% after the second stage of grinding. As a result, the activity index of biomass ash was enhanced from 102% to 112%, while the activity index of ceramic powder was kept constant at the age of 28 days. Regardless, due to a delayed pozzolanic reaction of fine CP particles over time, the activity index increased from 104% to 115% at the age of 90 days as a result of an additional grinding procedure.

Furthermore, the additional grinding of SCMs caused minor changes in their chemical composition, i.e., the transformation of the amorphous silica to crystalline form and vice versa. A lower amount of the amorphous silica was observed in the additionally milled ceramic powder sample (CP’), while a slight increase in reactive silica was registered in B2′. Resultantly, the biomass ash fell under the category of high pozzolanicity (Class 10). Despite the reduced reactive silica amount in CP, the subsequent grinding of the material led to the highest class (Class 15), signifying the superior pozzolanicity of this SCM type.

At last, the size-variable bulk inorganic matter in raw materials, i.e., the particle size of the raw, unprocessed biomass ash and ceramic waste, is surely the contributing factor to the efficiency of the employed grinding procedure.

The availability of a greater number of nucleation sites for the pozzolanic reaction to take place as a result of a higher specific area could be one of the reasons for the improvement in pozzolanic reactivity [18]. Another reason could be the filler effect of finer particles as it results in better pore refinement and higher compactness of the matrix [30].

#### 3.1.4. X-ray Powder Diffraction Technique Analysis

**Figure 1:**
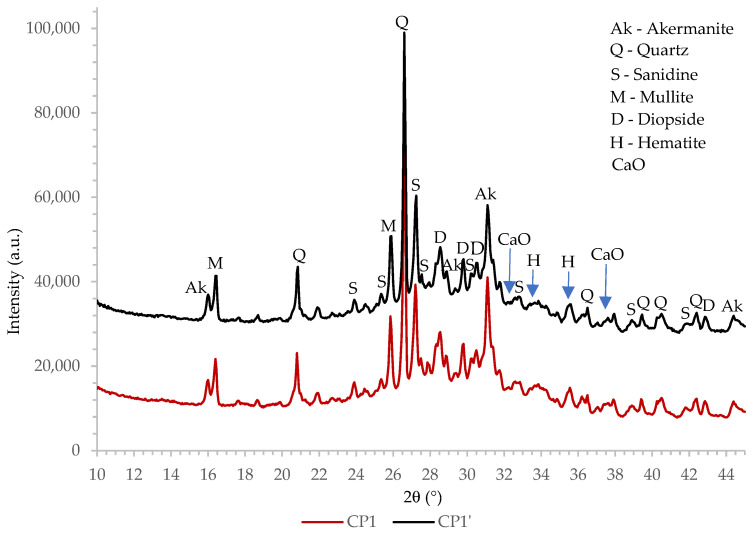
The XRD patterns of ceramic powder.

**Figure 2:**
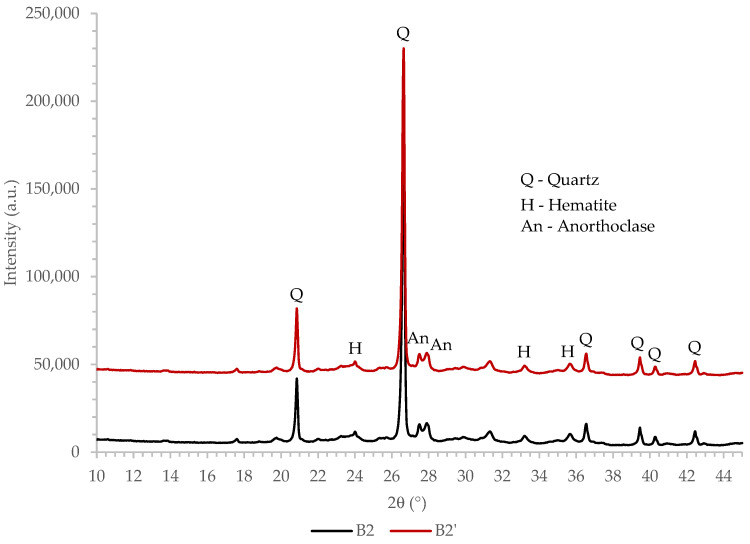
The XRD patterns of biomass ash.

The XRD patterns, collected on ceramic powder and biomass ash, were used for the determination of the mineralogical composition as well as for crystallinity studies, as presented in Figure 1 and Figure 2. As can be seen from Figure 1, a broad amorphous peak is present at the 2θ range of 20° to 36°, confirming the presence of an amorphous phase in samples CP1 and CP1′. The amorphous background width and height are almost identical for both CP1 and CP1′ materials, indicating that the fraction of the amorphous phase insignificantly changed during the mechanochemical activation in the vario-planetary mill. On the other hand, the presence of an amorphous phase was not clearly and unambiguously visible in the XRD patterns of biomass ash (Figure 2).

The mineralogical composition was determined by comparing the collected XRD patterns with the data from the Inorganic Crystal Structure Database (ICSD). Ceramic powders CP1 and CP1′ consist mainly of quartz, akermanite and sanidine. Other minerals identified in the ceramic waste are mullite and diopside. The presence of hematite and calcium oxide is also evident although the most intensive diffraction peaks do not rise significantly above the background. Hematite is commonly known, even in low concentrations, to be responsible for the reddish color of ceramics [31]. Considering XRD patterns of the biomass ash, quartz is the most abundant mineral. Other identified crystalline phases are hematite and anorthoclase.

Mechanochemical activation (high energy dry milling) may cause crystal structure deformation (crystallite size change and strain deformations), particle morphology changes and the amorphization of crystalline phases, which can be characterized by the XRD technique [32,33,34,35]. Moreover, mechanochemical activation can result in mechanochemical reactions and the appearance of new phases [36]. A detailed analysis of XRD patterns showed that the additional peaks were not registered within those of mechanically activated CP1′ and B2′, indicating that mechanochemical reactions did not occur during the milling process in the vario-planetary mill. Negligible differences in peak positions, their widths and intensities were also revealed in the XRD patterns of CP and CP1′ and in those of the B2 and B2′ samples. Those findings indicate that the lattice parameters and microstructure (crystallite size and strain) of crystalline phases insignificantly changed during the additional milling.

#### 3.1.5. Particle Size Distribution

**Figure 3:**
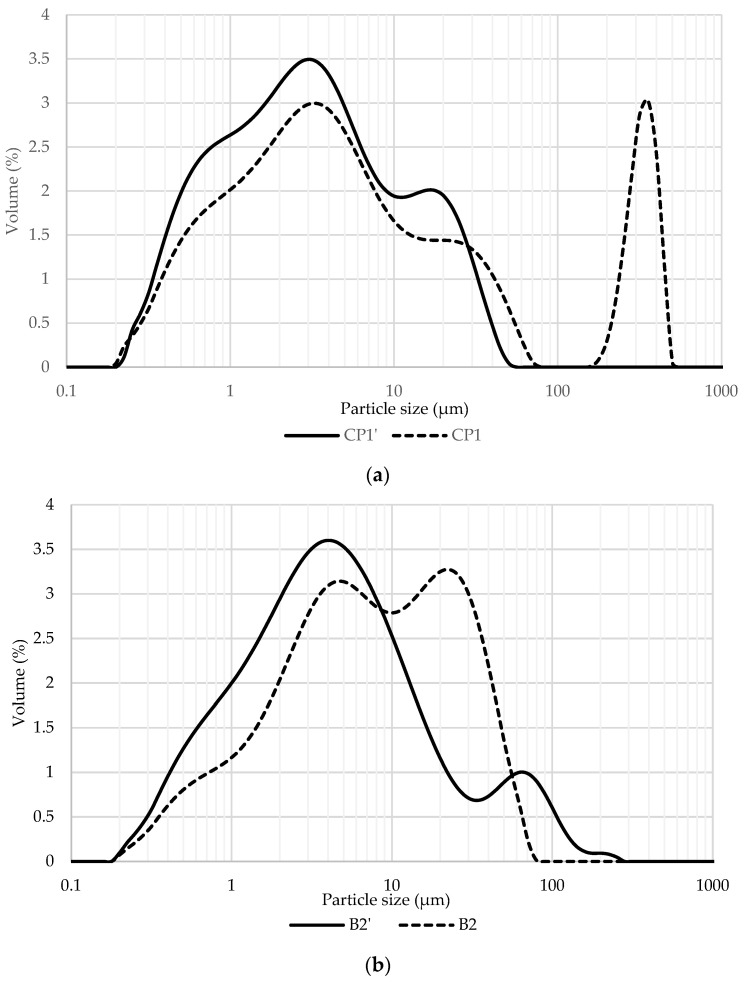

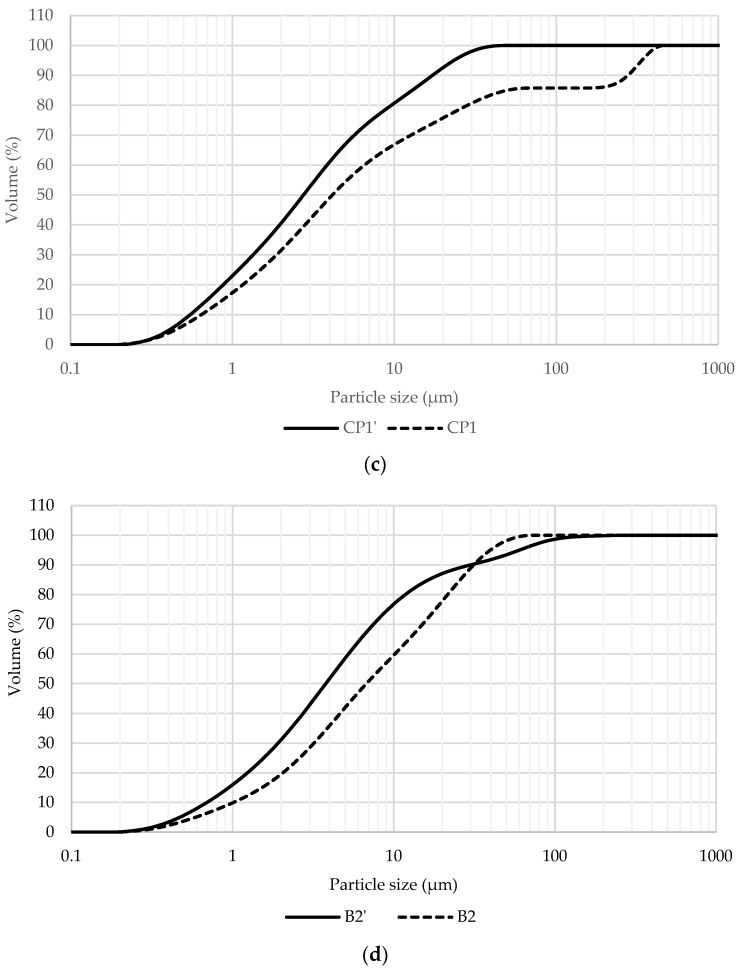
Volume-based frequency (**a**,**b**) and cumulative (**c**,**d**) particle size distribution curves of ceramic and biomass ash powders.

The analysis of particle size distribution is an important parameter for understanding the physical and chemical properties of a material. Volume-based particle size frequency distribution for ceramic waste CP1/CP1′ and biomass ash B2/B2′ are illustrated in Figure 3. The obtained particle size distribution (PSD) curves enabled an estimation of the dispersion degree of particles within the milled samples over the dimensional range from 20 nm up to 2 mm. All the investigated samples demonstrated bimodal particle size frequency distribution curves. The PSD curve for CP1 shows a significant portion of particles whose diameter is bigger than 100 μm. Such big particles were not detected in the PSD curve of the additionally milled CP1′ (see Figure 3a). It is also clearly evident that the percentage of the particles smaller than 30 μm is greater for sample CP1′ compared to that of CP1.

The graphs in Figure 3b illustrate the PSD of biomass ash. For the additionally milled B2′ sample, it is evident that the fraction of particles smaller than 8 μm is bigger than that of B2 as well as that the percentage of particles with the diameter between 10 and 50 μm is smaller. Nevertheless, the PSD curve of sample B2′ shows the presence of the particles larger than 80 μm whose fraction is negligible in sample B2. This could be explained by the fact that agglomeration/aggregation often occurs during a prolonged and more intensive milling process.

Cumulative undersize PSD curves are exhibited in Figure 3c,d. The value of the cumulative curve of the additionally milled samples CP1′ and B2′ is generally higher, at a certain particle size, indicating finer particles. An exception occurs for the particle size interval between 30 and 100 μm for the biomass ash sample, possibly due to the agglomeration of the particles.

Particle size distribution parameters are given in Table 7. The d(0.1), d(0.5) and d(0.9) values indicate particle diameters corresponding to 10%, 50% and 90% of the cumulative undersize distribution while D(4,3) is the volume-weighted mean. Although the median particle size diameter d(0.5) and volume-weighted mean are in the same order of magnitude, the mentioned parameters are smaller for the additionally milled CP1′ and B2′.

All findings indicate that additional milling had a significant effect on the particle size reduction of both ceramic powder and biomass ash materials. For instance, the median particle size diameters d(0.5) were reduced by 34% and 44% for the additionally milled samples CP1′ and B2′, respectively.

### 3.2. Properties of Mortars Blended with Selected SCMs

#### 3.2.1. Flexural Strength of Hardened Mortar

**Figure 4:**
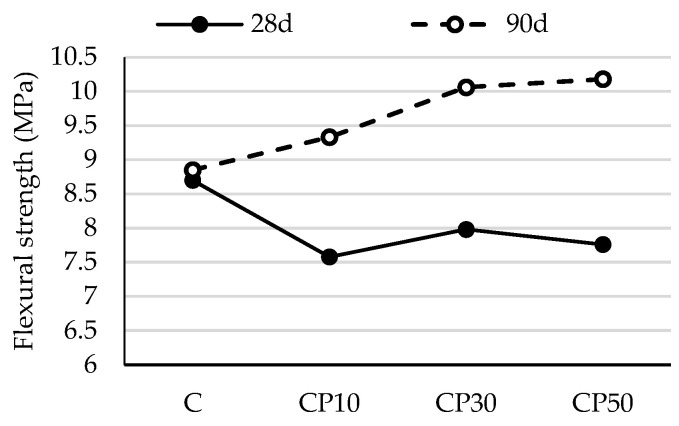
Flexural strength of mortars blended with ceramic powder.

**Figure 5:**
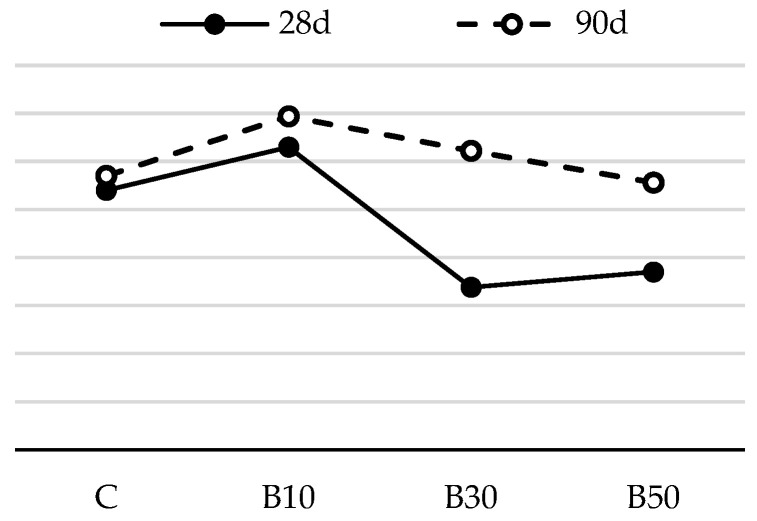
Flexural strength of mortars blended with biomass ash.

The flexural strengths of mortar samples after 28 and 90 days are shown in Figure 4 and Figure 5.

As shown in Figure 4, the flexural strength of the mortars blended with CP was lower than that of the reference mortar at 28 days. This is because its contribution to the strength of the mortar is not as substantial as that of PC early on. Over time, there was a steady strength increase in the mortars containing CP. All the mortars using CP attained a higher strength value than the reference mortar at 90 days. This may be attributed to the delayed pozzolanic reaction, as reactive silica consumes portlandite and produces more C-S-H gel, which increases the strength of mortar samples. A number of studies have published similar conclusions [12,37]. For instance, the flexural strength of CP10, CP30 and CP50 went down by 13%, 8% and 11%, respectively, when compared to that of the reference mortar at 28 days, while the 90-day strength of the blended mortars rose by 5%, 14% and 15% in comparison with the reference mix, respectively.

The results presented in Figure 5 show that, among biomass ash-blended mortars, the mix containing 10% biomass ash displays the highest flexural strength at the age of 28 days. At this stage of hydration, this strength increase may be attributed to the packing effect of fine B2 particles. When this amount of replacement is exceeded, there is a slight loss in strength, which may reach as low as 90% of the strength of the reference mortar. Similar to the influence of CP, it is notable that the flexural strength of the mortar containing B2 improves at later ages, as additionally formed C-S-H gel partially fills the gaps and/or voids between cement clinkers and physically densifies the granular compactness [38]. As a result, mortar with 50% biomass ash has a comparable flexural strength with the reference mortar at 90 days.

The substitution of cement by the selected SCMs, regardless of the SCM type and replacement level, did not have a detrimental effect on mortar’s flexural strength. Assuming that the pozzolanic reaction had not been intensified up to the age of 28 days, such negligible effect can be attributed to: (1) the increase in nucleation sites for cement hydration (provided by the higher fineness of the SCM), resulting in more evenly dispersed reaction products; this accelerates the cement hydration process and results in a faster early-age strength gain; or (2) the filler effect: fine SCM particles act as micro aggregates, filling the voids in the binder matrix and enhancing the compactness of the mortar structure.

#### 3.2.2. Compressive Strength of Hardened Mortar

In most cases, a correlation can be seen between the flexural strength of mortar and its compressive strength. In light of this, it may be deduced that the compressive strength likewise improves as the flexural strength increases.

**Figure 6:**
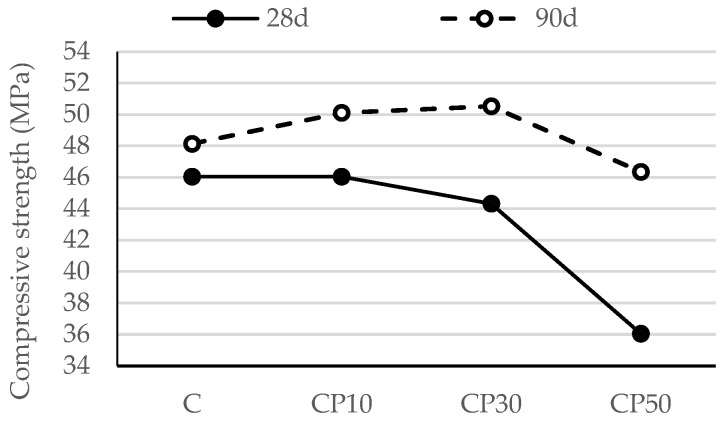
Compressive strength of mortars blended with ceramic powder.

As discussed in Section 3.2.1, the compressive strength of mortars blended with CP (Figure 6) followed a trend similar to that of mortars’ flexural strength at the age of 28 days. As the replacement level rose, fewer cement particles were available for hydration, which decreased the number of hydration products and adversely affected the compressive strength, porosity and the permeability of the mix due to the dilution effect. This finding is in agreement with the experimental studies conducted by Santosh et al. [39], Ohemeng et al. [40] and Raheem and Ikotun [41]. As a result, cement replacement of 50% led to a decrease in the compressive strength of the reference sample by 22%—Figure 3. At later ages, the compressive strength gain of the mortar blended with CP was mainly brought about by the pozzolanic reaction. Some authors underline the improved availability of more nucleation sites for the reaction as an additional reason for the rise in compressive strength [42]. Hence, at the age of 90 days, CP50 demonstrated a compressive strength comparable to the reference sample, while CP10 and CP30 exceeded that of the reference one.

**Figure 7:**
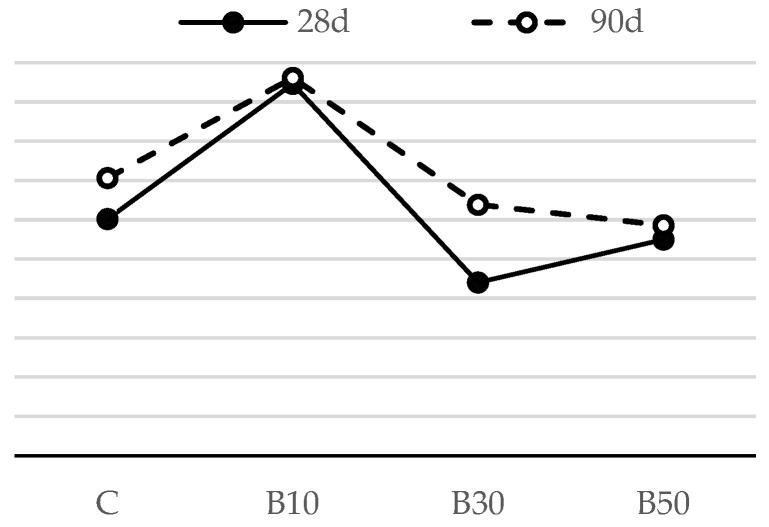
Compressive strength of mortars blended with biomass ash.

Figure 7 presents the compressive strength test results for the biomass ash–blended mortar mixtures after 28 and 90 days of curing. It can be observed that no significant strength drop occurred with an increase in the cement substitution level. Clearly, there was a slight decline in the compressive strength of the mortar when B2 was used at the levels up to 30%. Nevertheless, this difference was minimized as the curing age advanced. Regardless of the age, 10% cement replacement resulted in an increase in compressive strength values, while, at the age of 90 days, B30 and B50 achieved the strengths comparable to the reference mortar. A similar finding on the inclusion of this type of biomass ash was also reported by Adesanya [43], also stating that there is no significant difference between the strength of the mortar produced with 0.0 and that of the one containing 20.0% corn cob ash.

#### 3.2.3. Capillary Water Absorption of Hardened Mortar

**Figure 8:**
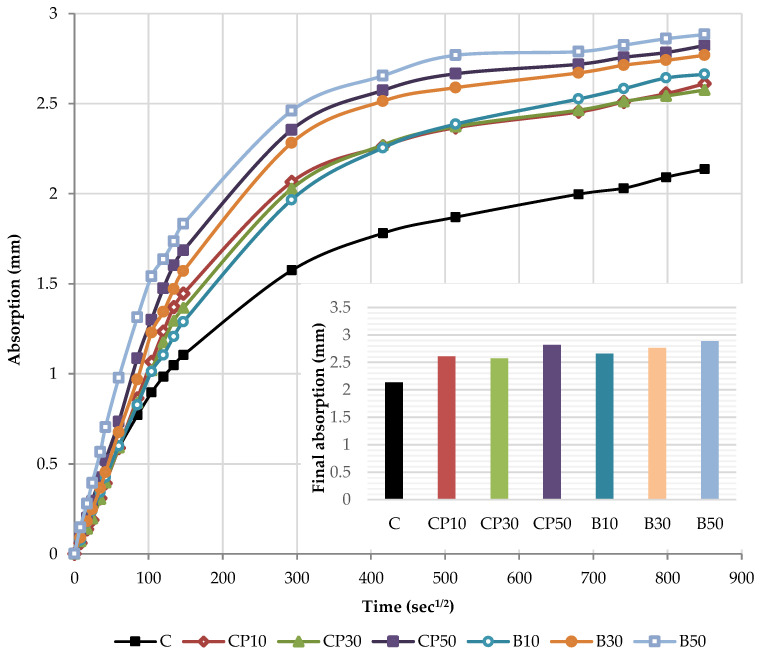
Capillary water absorption: the kinetics and the final values.

The capillary water absorption of all the mortars prepared with varying contents of the selected SCMs was tested after the curing period of 28 days and capillary absorptivity coefficients were determined. The kinetics of capillary water absorption and the final absorption values for all the mortars prepared with varying contents of the selected SCMs are demonstrated in Figure 8.

The water absorption rose as the cement replacement level increased, regardless of the SCM type and measured time. Considering the final values, a notable rise in the absorption of 22%, 21% and 32% was recorded for CP-based mortars at 10%, 30% and 50% replacement levels, respectively. Similarly, the inclusion of biomass ash: B10, B30 and B50 gradually increased the water absorption by 25%, 30% and 35% compared to the reference mortar, respectively. This behavior is associated with a higher pore volume of the mortar as SCM content increases, influenced by the dilution effect. The reduction in the cement content increases the amount of free water intended to react with cement particles and leads to a higher effective w/b, correspondingly, as presented in Table 1. This observation has been confirmed in many studies [39,40,44]. A higher w/b results in a greater capillary porosity and adversely affects the absorption of the SCM blends.

The cumulative water absorption process of mortars increases nonlinearly with the square root of time. According to the growth trend, it is divided into two stages: (1) initial absorption (Si), between 1 min and 6 h, mainly related to water diffusion in the macro pores and (2) secondary absorption (Ss), between one and eight days, predominantly associated to water diffusion in small pores. The water uptake has a good linear relationship with the square root of time t^1/2^.

In the initial stage, the slope of the curve is the largest, and the growth rate is the fastest owing to a high absorption capacity of macro pores. As the macro pores are gradually saturated, the water diffusion in the smaller pores is slowed down, the capillary water absorption capacity gradually decreases and the water absorption rate curve gradually levels off.

The kinetics of the capillary absorption of water into the mortars can be described by the capillary absorptivity coefficients, which are defined as the slopes of the fitted curves.

**Figure 9:**
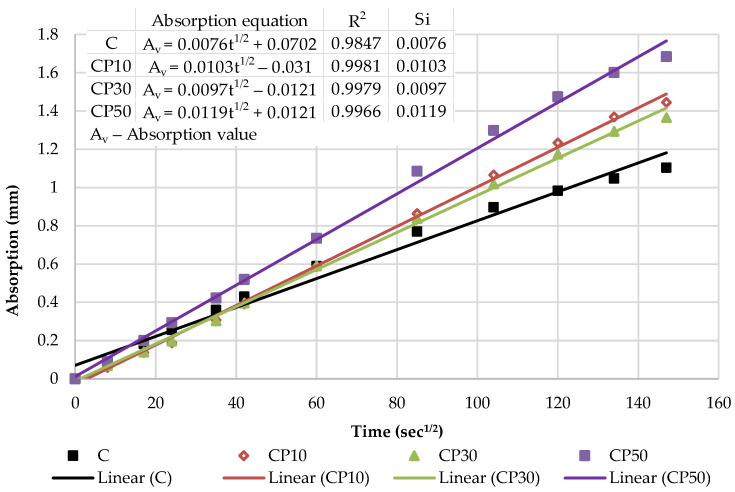
The initial capillary absorptivity of CP–based mortars.

**Figure 10:**
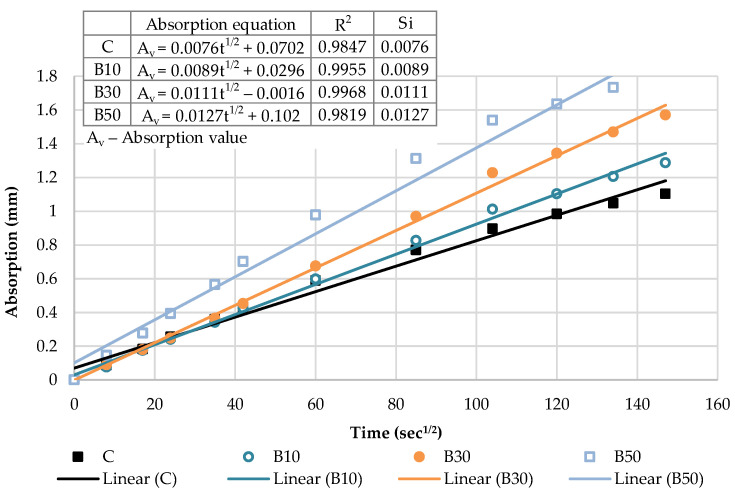
The initial capillary absorptivity of biomass ash–based mortars.

Figure 9 and Figure 10 show the initial absorptivity calculated as the slope of the absorption vs. the square root of time during the first 6 h of the test. Absorptivity values are documented for all mixtures, as the correlation coefficients are equal to or higher than 0.98. All blended mortar mixes showed a higher initial capillary absorptivity coefficient (Si) in relation to the reference mortar. The mortars with the highest replacement level—CP50 and B50—displayed the highest water absorption rates during the whole testing process, whereas Si rose by 57% and 67% compared to the reference sample, respectively.

**Figure 11:**
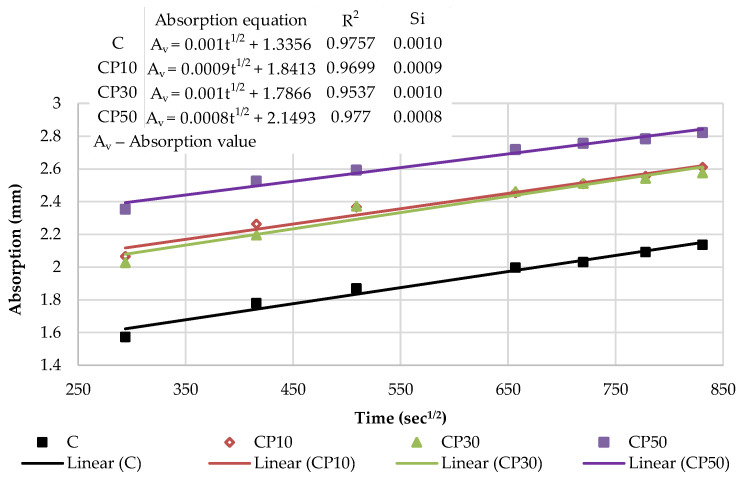
The secondary capillary absorptivity of CP–based mortars.

**Figure 12:**
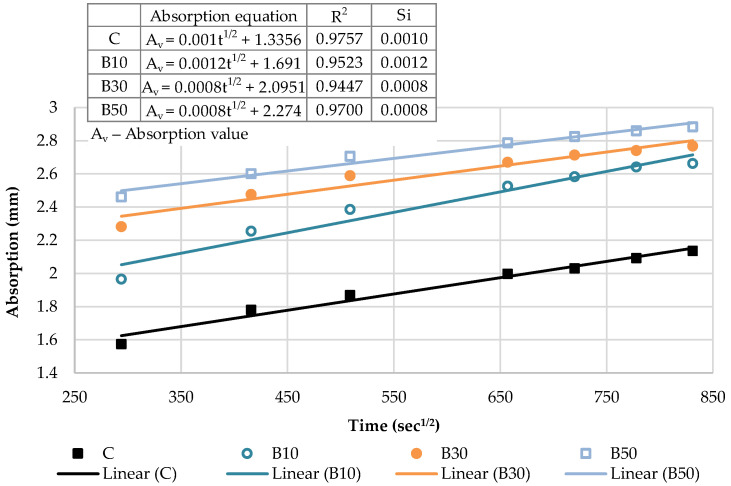
The secondary capillary absorptivity of biomass ash–based mortars.

Figure 11 and Figure 12 illustrate the secondary absorptivity calculated as the slope of the absorption vs. the square root of time between one and eight days of testing. The absorptivity increases linearly with the square root of time; hence, the correlation coefficients are equal to or higher than 0.98. It can be noticed that there is a considerable difference in the total amount of water absorbed by the samples in the two different stages. The inclusion of SCMs leads to an only slight variation in the secondary capillary absorptivity coefficients (Ss). CP50 and B50 are characterized by the lowest Ss, whereas the absorption values decreased by 20% compared to the reference mortar. This may be explained by the high initial absorption of these samples. During this initial absorption, it can be assumed that—since most of the water was already absorbed in the first hours of the test—the secondary rate of absorption will be much lower. A similar finding was reported by Caestro et al. [45].

### 3.3. Properties of Mortars with Decreased w/b Ratio

Utilizing additionally ground materials could be effective when improving the properties of blended mortar, as demonstrated in Section 3.1.3. However, grinding time is directly related to energy consumption. Hence, it is imperative to note that further grinding requires higher energy demand, which is uneconomical. Therefore, the authors chose to explore the possibility of improving the properties of the mortars blended with the selected SCMs by decreasing w/b ratio instead of additionally grinding these materials.

Based on the results presented in the previous Chapter, it can be stated that the addition of SCMs can modify the hydration rate and products, which influences the permeability of cement–based materials, especially at early ages. Lowering w/b is a conventional method for reducing the permeability of cement-based composites, hence enhancing their mechanical and durability properties. A lower w/c ratio may lead to reduced porosity as well as smaller pore sizes, which contributes to a denser structure with better mechanical properties [27]. Therefore, in order to enhance impermeability, decrease capillary porosity and reduce water absorption, the mortars with a lowered effective w/b were cast and, at the appropriate age, tested. The results are listed hereinafter.

#### 3.3.1. Flexural Strength of Hardened Mortar with Decreased w/b

**Figure 13:**
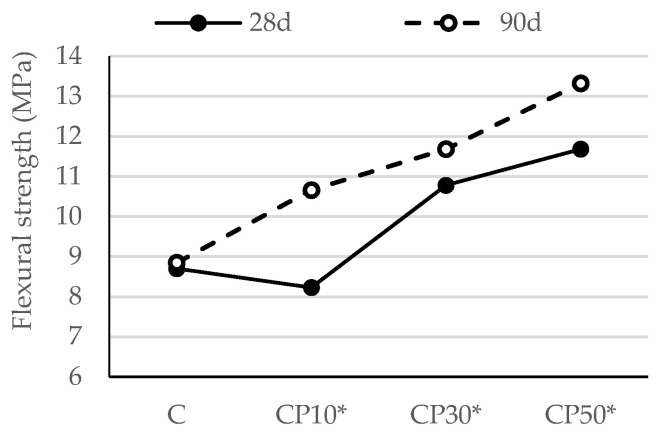
Flexural strength of mortars blended with ceramic powder, with decreased w/b.

**Figure 14:**
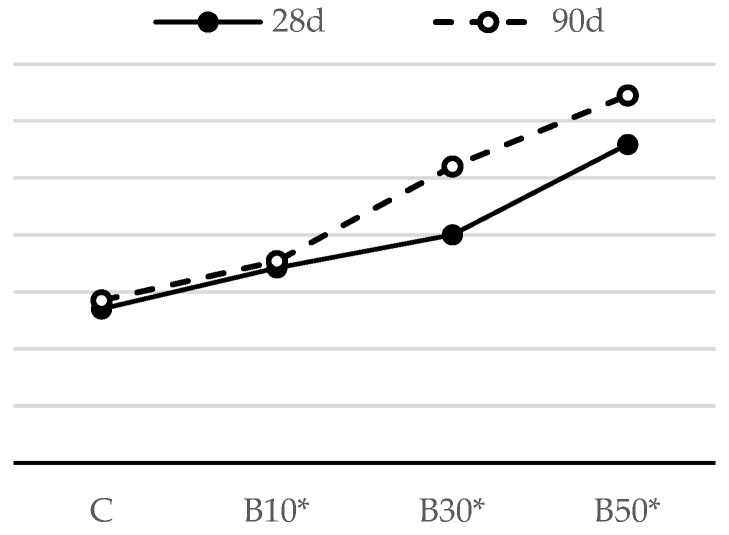
Flexural strength of mortars blended with biomass ash, with decreased w/b.

Figure 13 and Figure 14 show the flexural strength of the reference cement mortar and the mortars blended with the selected SCMs with a decreased w/b ratio.

The experimental results showed that, regardless of the SCM type, increasing the replacement level resulted in a noteworthy increase in the flexural strength of the mortar with a decreased w/b ratio. Hence, a lower w/b effectively reduced the mortar’s porosity, densified the matrix and increased the mortar’s flexural strength.

Compared to the reference mortar, the flexural strength of the CP30* and CP50* samples improved by 24% and 34% (Figure 13), respectively, while the flexural strength of B10*, B30* and B50* followed a similar trend and displayed an improved capacity by 15%, 22% and 33%, respectively, at the age of 28 days (Figure 14). This outcome is a clear result of a declining porosity, greater compactness and, consequently, enhanced mechanical properties. These results are in agreement with the findings from previous studies [46]. A slight decline of 5.4% in flexural strength is recorded for the mortar containing CP as a replacement of 10% PC compared to the reference mortar. This decline occurred owing to the dilution effect, i.e., a decreased number of hydration products at the early stage of hydration, which has a dominance over the negligible difference in w/b between this type of mortar and the reference one.

As expected, there was a sharp strength increase in the mortar using SCMs at 90 days; hence, all the mortars using CP and B2 attained the strength higher than that of the reference sample. These results are related to the pozzolanic properties of SCMs, which contribute to strength development in the long term. The rise in flexural strength was induced by the formation of secondary C-S-H gel, which is responsible for the enhanced microstructure of mortar. Similar findings were also reported on the inclusion of other SCMs [47,48].

#### 3.3.2. Compressive Strength of Hardened Mortar with Decreased w/b

**Figure 15:**
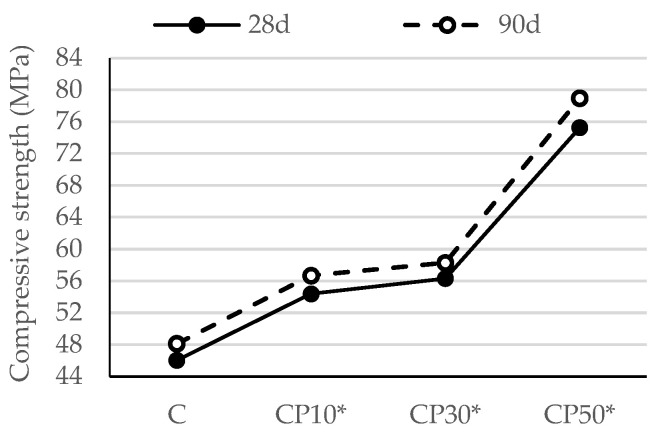
Compressive strength of mortars blended with ceramic powder, with decreased w/b.

**Figure 16:**
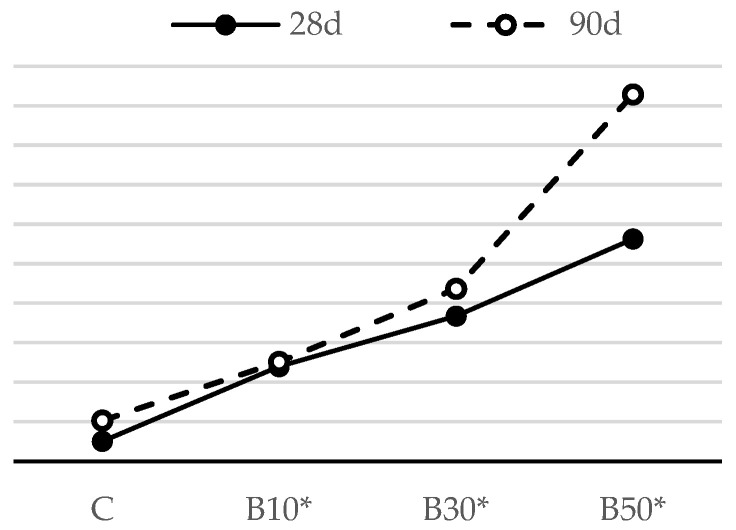
Compressive strength of mortars blended with biomass ash, with decreased w/b.

Figure 15 and Figure 16 illustrate the influence of a decreasing w/b on the compressive strength of the mortar blended with the selected SCMs.

The compressive strength results show a similar development in trend as that of flexural strength. At the age of 28 days, the compressive strength of the CP10*, CP30* and CP50* samples rose by 18%, 22% and 63% in relation to the reference mortar, respectively, while the compressive strength of B10*, B30* and B50* improved by 16%, 27% and 44%, respectively.

These effects are ascribed to the refinement of the pore structure and densification of the matrix with the decreasing w/b. This observation is consistent with the findings in [28,39,49,50]. The diminished pore spaces induced by the lower w/b result in the removal of larger voids and the formation of tiny unconnected pores, thereby contributing to the density increase. Some authors indicated that the interfacial bonding strength increases with a decreasing w/b, making samples more difficult to crack [51,52]. Furthermore, according to the findings of some authors [53,54], these tiny unconnected pores can effectively slow down the penetration of harmful chemicals (e.g., carbon dioxide, chloride and sulfur ions), thus reducing the deterioration rate of cement-based composites and the steel reinforcements inside.

A comparison of compressive strength results of the same mortar samples cured for 28 and 90 days reveals a growth in these values for the longer-cured samples, on account of a prolonged curing period and a delayed pozzolanic reaction. The pozzolanic reactions produced additional C-S-H gel and led to a reduction in voids and an enhancement in the compressive strength over time. The synergistic effect of a decreased w/b and the pozzolanicity of the selected SCMs are particularly noticeable at higher cement replacement levels, as pozzolana improved the compressive strengths of mortars CP50* and B50* by 69% and 64% in relation to the reference mortar, respectively.

However, it can be noted that all mortars, apart from B50*, achieved most of their compressive strength values up to the age of 28 days of curing. A lower w/b ratio implies: (1) a smaller amount of water in the concrete mix, (2) a pore refinement due to the reduced interparticle distance, (3) a transition of the capillary to finer pores and (4) the densification of the mixture where the continuous consumption of free water is limited. Owing to these implications, it may be assumed that pozzolanic reactivity cannot be developed efficiently up to the age of 90 days. A complete hydration of cement generally only requires a w/c ratio of 0.4. Beyond this value, extra water is not necessary for hydration, but only for a delayed pozzolanic reaction. A lower w/b allows cement to consume all the available water and hydrate up to the age of 28 days, inhibiting or at least slowing down the reaction between portlandite, reactive silica and water afterward. As a result, the strength gains over time are significantly lower than those of the mortars with a higher effective w/b, which was the case in this research. Zhao et al. [39] underlined a similar conclusion and stated that the filling effect and secondary hydration effect of pozzolana at low w/b are weaker.

#### 3.3.3. Capillary Water Absorption of Hardened Mortar with Decreased w/b

**Figure 17:**
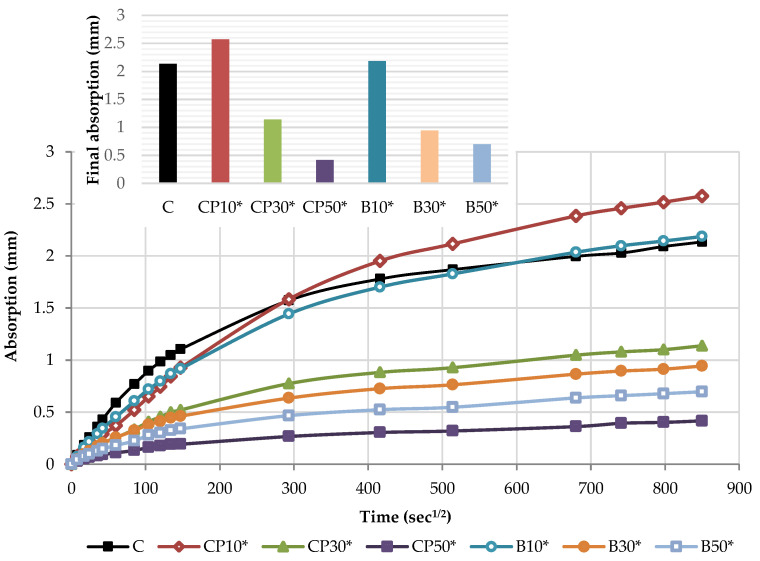
Capillary water absorption of mortars with decreased w/b: the kinetics and the final values.

Figure 17 presents the kinetics of capillary water absorption and the final absorption values for the reference mortar and blended mortars with a decreased effective w/b, after 28 days of curing.

As depicted in Figure 17, decreasing w/b had a significant influence on the mortars blended with the selected SCMs. In the initial period, as w/b ratio decreased, all the blended mortars exhibited a slower water absorption rate and a smaller water absorption capacity, thus suggesting a denser structure. A denser mortar structure, as reported by Wang et al. [52], corresponds to a denser and stiffer surface exposed to water and, consequently, a slower absorption rate. Considering the final absorption values, only the mortars with 10% cement replacement showed higher absorption values in relation to the reference mix. A lower w/b was found to induce a significantly denser structure of the mortars blended with a high volume of SCMs (30 and 50%). The absorption results of blends CP30*, CP50*, B30* and B50* declined by 47%, 80%, 56% and 68% compared to the reference sample, respectively.

**Figure 18:**
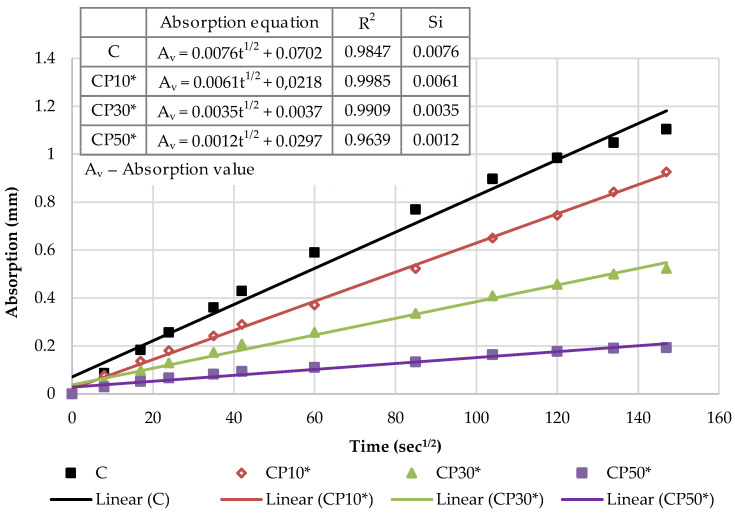
The initial capillary absorptivity of CP–based mortars with decreased w/b.

**Figure 19:**
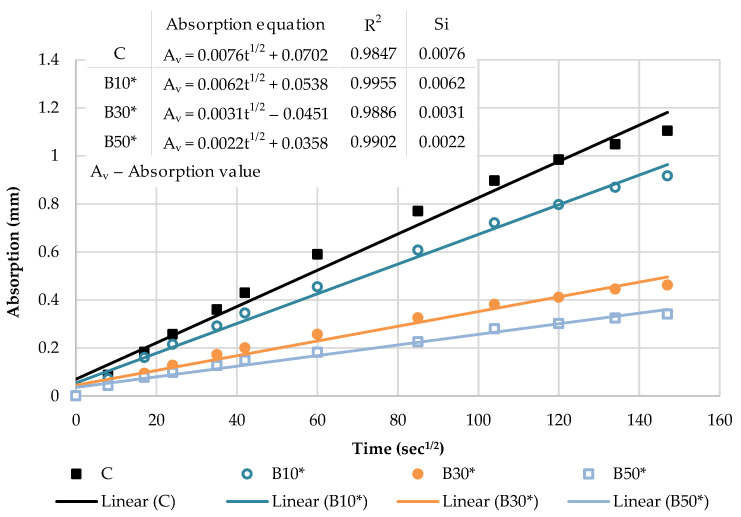
The initial capillary absorptivity of biomass ash–based mortars with decreased w/b.

Figure 18 and Figure 19 show the initial absorptivity calculated as the slope of the absorption vs. the square root of time during the first 6 h of the test. The absorptivity increases linearly with the square root of time and the correlation coefficients are equal to or higher than 0.98; hence, the initial capillary absorptivity coefficient can be determined. All blended mortars showed a lower Si in relation to the reference mortar, which is attributed to the denser structure of the mortar with a decreased w/b and diminished capillary pores. In other words, the water infiltration resistance of all of the mixes in the initial period was improved.

**Figure 20:**
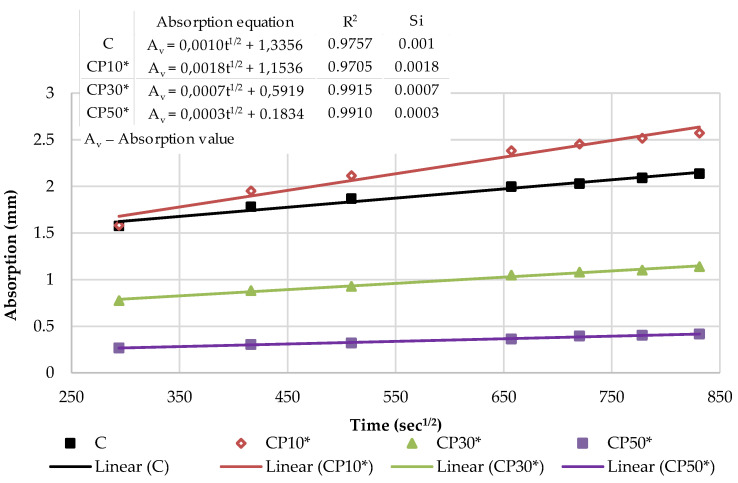
The secondary capillary absorptivity of CP–based mortars with decreased w/b.

**Figure 21:**
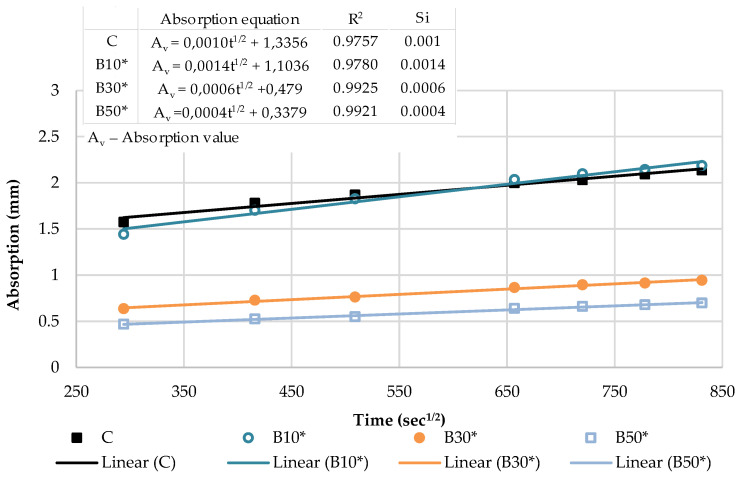
The secondary capillary absorptivity of biomass ash–based mortars with decreased w/b.

Figure 20 and Figure 21 illustrate the secondary absorptivity calculated as the slope of the absorption vs. the square root of time between one and eight days of testing. The absorptivity increases linearly with the square root of time for all mortar blends. The increased rate indicates a slow-growth stage and has a tendency to stabilize, which means that the capillary water absorption capacity gradually decreases and the water absorption rate curve gradually levels off. It can be observed that Ss increased slightly with 10% cement replacement levels. Contrastingly, as the cement substitution rate increases, effective w/b decreases and the Ss of mortar blends shows a decreasing trend. It can be assumed that most of the water was already absorbed in the initial absorption period of the blends with a high volume of SCMs (30% and 50%); hence, the secondary rate of absorption is noticeably lower. Compared with the reference mortar, the Ss of CP30* decreased by 30%, CP50* dropped by 70%, B30* was reduced by 40% and B50* declined by 60%.

The obtained results confirm lowering w/b as a long-standing method for reducing the porosity of cement–based composites, which contributes to a denser matrix structure with a lower permeability and a higher compressive strength. These outcomes are favored in major infrastructure projects—such as long–span bridges—and in aggressive environments, where a lower w/b can reduce the diffusion rate of chlorides and, thereby, minimize the corrosion risk of internal steel reinforcements.

## 4. Conclusions

The presented comprehensive study suggests that the selected industrial and agricultural by-products can be effectively used as SCMs in large amounts for the production of sustainable blended cement mortar. The key conclusions that arise from the study are given below:

- The chemical analysis showed that finely ground ceramic powder and corn cob-based biomass ash can be used as pozzolanic materials owing to a relatively high amorphous silica content and satisfactory level of fineness.
- The effect of the grinding procedure has a significant influence on the microstructural and pozzolanic properties of the tested SCMs. Additional grinding improved the pozzolanicity of materials, as a consequence of: (1) the partial conversion of crystalline to amorphous silica, (2) the availability of a greater number of nucleation sites for the pozzolanic reaction to take place, (3) the better pore refinement and higher compactness of the matrix due to the filler effect.
- Despite filler and nucleation effects, a higher replacement level decreased the content of cement clinker and the hydration products, which further increased the capillary porosity and permeability of the mortar (the dilution effect) resulting in lower compressive and flexural strengths and greater water absorption. These effects were particularly noticeable at the early stage of hydration (28 days of curing) when the pozzolanic reaction was not developed, bringing about an increase in the free water content intended to react with cement particles.
- In light of the reported effects of the water-to-binder ratio, it is evident that the overall performance of the tested mortars has improved, once again proving this method is an effective conventional technique to create more durable cement-based composites. The compressive strength of ceramic powder-blended mortars (CP10*, CP30* and CP50*) rose by 18%, 22% and 43%, while the strength of corn cob ash-blended mortars (B10*, B30* and B50*) improved by 16%, 27% and 44%, in relation to the reference mortar, respectively. Owing to the reduced permeability, the capillary water absorption of the blends with the highest SCM content (B50* and CP50*) declined by 80% and 68% compared to the reference mix, respectively.

The outcome of the conducted experiment draws a conclusion that lowering w/b is beneficial for mortar by mitigating the dilution effect, i.e., reducing porosity and enhancing mechanical properties. Therefore, finely ground ceramics and harvest residue ashes may be considered a viable alternative to mainstream SCMs, such as fly ash or granulated blast-furnace slag at higher PC replacement levels in cement–based composites.

The reutilization of agricultural residues and industrial by-products in the cement industry serves a pivotal role in relieving the environmental burden by providing an effective method of waste management that excludes open-field burning or landfills along with reducing greenhouse gas emissions as the need for raw material extraction for cement clinker manufacture is also minimized in this way.

## Figures and Tables

**Table 1:** Labels and component materials for mortars and the effective w/b.

Mortar	m_c_ (g)	m_scm_ (g)	m_s_ (g)	m_w_ (g)	eff. w/b
C	450	/	1350	225	0.5
B10	405	45	1350	225	0.532
B30	337.5	112.5	1350	225	0.631
B50	225	225	1350	225	0.884
CP10	405	45	1350	225	0.532
CP30	337.5	112.5	1350	225	0.631
CP50	225	225	1350	225	0.884

m_c_—mass of cement; m_scm_—mass of SCM; m_s_—mass of sand; m_w_—mass of water; eff. w/b–effective water-to-binder ratio.

**Table 2:** Labels and component materials for mortars and the effective w/b.

Mortar	m_c_ (g)	m_scm_ (g)	m_s_ (g)	m_w_ (g)	eff. w/b	m_hrwr_ (g)
B10 *	405	45	1350	211.5	0.5	2.5
B30 *	337.5	112.5	1350	178.3	0.5	4.5
B50 *	225	225	1350	133.3	0.5	12.0
CP10 *	405	45	1350	211.5	0.5	2.0
CP30 *	337.5	112.5	1350	178.3	0.5	5.0
CP50 *	225	225	1350	133.3	0.5	13.0

m_c_—mass of cement; m_scm_—mass of SCM; m_s_—mass of sand; m_w_—mass of water; eff. w/b–effective water-to-binder ratio; m_hrwr_—mass of superplasticizer; *—mortars with a decreased effective w/b; 10,30,50—cement replacement levels.

**Table 3:** Chemical composition of SCMs.

	PC	CP1	CP2	CP3	B1	B2
Loss on ignition at 950 °C, %	/	3.30	0.30	1.00	3.60	2.40
SiO_2_, %	17.34	60.86	61.88	59.03	20.21	45.76
Al_2_O_3_, %	4.53	16.38	16.46	15.81	1.83	5.92
Fe_2_O_3_, %	20.64	6.81	7.40	6.64	1.74	3.38
Na_2_O, %	0.20	0.77	1.63	1.5	0.00	0.00
K_2_O, %	0.59	2.39	2.81	2.5	23.09	13.10
MgO, %	1.93	3.89	3.66	4.2	8.30	8.30
CaO, %	50.26	9.38	4.90	5.72	13.42	14.08
SO_3_, %	3.06	0.80	0.08	0.07	2.88	1.26
P_2_O_5_, %	0.00	0.14	0.20	0.16	7.78	2.81
Cl^−^, %	0.00	0.002	0.003	0.000	0.338	0.502
Reactive SiO_2_, %	/	50.26	31.32	48.01	18.78	35.19
Free CaO, %	/	6.45	4.69	5.65	5.96	11.34

**Table 4:** Chemical composition—criteria fulfillment.

	Chemical Requirements (EN 450-1)	Criteria	Standard	CP1	CP2	CP3	B1	B2
Chemical properties	Total amount of oxides: SiO_2_ + Al_2_O_3_ + Fe_2_O_3_ (%)	≥70%	EN 196-2 EN 450-1	Yes 84.05	Yes 85.74	Yes 81.48	No 23.78	No 55.05
	Free CaO content (%)	≤1.5%	EN451-1 EN 450-1	No	No	No	No	No
	Reactive SiO_2_ content (%)	≥25%	EN 197-1 EN 450-1	Yes	Yes	Yes	No	Yes
	Loss of ignition (%)	A: Max 5% B: Max 7% C: Max 9%	EN 196-2 EN 450-1	A	A	A	A	A
	Chloride content (%)	≤0.1%	EN 196-2 EN 450-1	Yes	Yes	Yes	No	No
	Sulfate content (%)	≤3%	EN 196-2 EN 450-1	Yes	Yes	Yes	Yes	Yes
	Total amount of alkalis (%) Na_2_O + 0.658 K_2_O	≤5%	EN 196-2 EN 450-1	Yes	Yes	Yes	No	No
	Phosphate content (%)	≤5%	ISO 29581-2 EN 450-1	Yes	Yes	Yes	No	Yes

**Table 5:** Physical and pozzolanic properties of tested materials.

	Criteria	Standard	CP1	CP2	CP3	B1	B2
Specific gravity (g/cm^3^)	/	SRPS B.B8.032	2.62	2.61	2.59	2.36	2.44
Specific surface area (cm^2^/g)	/	EN 196-6	13815.0	11064.0	6200.0	8120.0	8090.0
Pozzolanic activity	Class 5: f_c_ ≥ 5 MPa f_fl_ ≥ 2 MPa Class 10: f_c_ ≥ 10 MPa f_fl_ ≥ 3 MPa Class 15: f_c_ ≥ 15 MPa f_fl_ ≥ 4 MPa	SRPS B.C1.018	f_c_ = 11.61 f_fl_ = 3.42 Class 10	f_c_ = 10.05 f_fl_ = 3.18 Class 10	f_c_ = 7.08 f_fl_ = 2.61 Class 5	f_c_ = 6.20 f_fl_ = 2.31 Class 5	f_c_ = 8.65 f_fl_ = 3.50 Class 5
Activity index	AI_28_ ≥ 75% AI_90_ ≥ 85%	EN 450-1	Yes	Yes	Yes	No	Yes
			AI_28_ = 100%	AI_28_ = 90%	AI_28_ = 90%	AI_28_ = 68%	AI_28_ = 102%
			AI_90_ = 104%	AI_90_ = 107%	AI_90_ = 98%	AI_90_ = 79%	AI_90_ = 115%
Initial setting time (min)	≥60	EN 196-3	Yes 160	Yes 160	Yes 165	No 25	Yes 165
		EN 197-1					
		EN 450-1					
Final setting time (min)	≤2 times the setting of cement	EN 196-3	Yes 220 ≤ 2 × 190	Yes 210 ≤ 2 × 190	Yes 225 ≤ 2 × 190	Yes 45 ≤ 2 × 190	Yes 285 ≤ 2 × 190
		EN 197-1					
		EN 450-1					
Soundness (mm)	≤10	EN 196-3	Yes 0.6	Yes 0.5	Yes 0.5	Yes 0.6	Yes 1.0
		EN 450-1					

**Table 6:** Chemical, physical and pozzolanic properties of selected ceramic powder and biomass ash.

	Criteria	CP1	CP1′	B2	B2′
Reactive SiO_2_, %	≥25%	50.26	47.27	35.19	39.35
Pozzolanic activity	Class 5: f_c_ ≥ 5 MPa, f_fl_ ≥ 2 MPa Class 10: f_c_ ≥ 10 MPa, f_fl_ ≥ 3 MPa Class 15: f_c_ ≥ 15 MPa, f_fl_ ≥ 4 MPa	f_c_ = 11.61 f_fl_ = 3.42 Class 10	f_c_ = 15.21 f_fl_ = 4.32 Class 15	f_c_ = 8.65 f_fl_ = 3.50 Class 5	f_c_ = 10.99 f_fl_ = 3.52 Class 10
Specific surface area (cm^2^/g)	/	13,815.0	14,958.0	8090.0	10,921.0
Activity index	AI_28_ ≥ 75% AI_90_ ≥ 85%	AI_28_ = 100% AI_90_ = 104%	AI_28_ = 100% AI_90_ = 115%	AI_28_ = 102% AI_90_ = 115%	AI_28_ = 112% AI_90_ = 120%

**Table 7:** Particle size distribution parameters.

	CP1	CP1′	B2	B2′
d(0.1) (μm)	0.72	0.61	1.13	0.77
d(0.5) (μm)	4.64	3.08	7.58	4.24
d(0.9) (μm)	310.34	19.24	35.07	33.44
D(4,3) (μm)	57.12	6.65	13.60	12.87

## Data Availability

Not applicable.
